# Fostering intergenerational communication through games: an exploratory study on metaphorical life games in family settings

**DOI:** 10.3389/fpubh.2025.1532603

**Published:** 2025-05-14

**Authors:** Ming-xi Sun, Zhi-feng Zhao, Huan Liu

**Affiliations:** School of Arts, Soochow University, Suzhou, China

**Keywords:** thematic analysis, family generations, life review, intergenerational communication, metaphorical play

## Abstract

**Introduction:**

Existing research indicates that life review is a crucial means of enhancing intergenerational communication. However, how to effectively facilitate this process through gamification in family settings remains unclear. This study explores how metaphorical life games can promote communication between older adults and adolescents during life review activities, highlighting the relationship between dialogue themes and game design elements that encourage meaningful interaction.

**Methods:**

We played a commercial game by recruiting 24 pairs of family members to play together. Younger partners asked older participants about their relevant memories during the game. The experimental data were analyzed inductively based on thematic analysis.

**Results and discussion:**

This study identified the effectiveness of life review through metaphorical games in facilitating communication in intergenerational family relationships by three core mechanisms: communication climate creation, symmetrical intergenerational interaction, and metaphorical expression elaboration. Intergenerational family engagement in metaphorical life games can effectively improve mutual communion and provide game design inspiration for digital games that support intergenerational life review. These findings offer new insights into life metaphor game design strategies and the mechanisms that enhance intergenerational communication.

## 1 Introduction

In the context of global aging and increased longevity, intergenerational communication has emerged as a critical public health priority, particularly as multigenerational families become more prevalent (1). Effective communication between older adults and younger generations is essential for fostering emotional wellbeing, reducing social isolation, and preserving cultural heritage (2). However, existing approaches to facilitating such dialogue—particularly through life review interventions—often rely on structured, therapist-led sessions or trained family members, limiting their accessibility and spontaneity in everyday family settings. While life review, defined as the intentional reflection on life experiences, has demonstrated benefits for mental health and intergenerational bonding, its reliance on formalized frameworks and unequal participation dynamics poses significant barriers to widespread adoption (3). This gap is further exacerbated by the rapid technological divide between generations, where older adults may struggle with digital tools that younger generations take for granted, creating asymmetrical interactions that hinder reciprocal communication (4).

**Figure 1:**
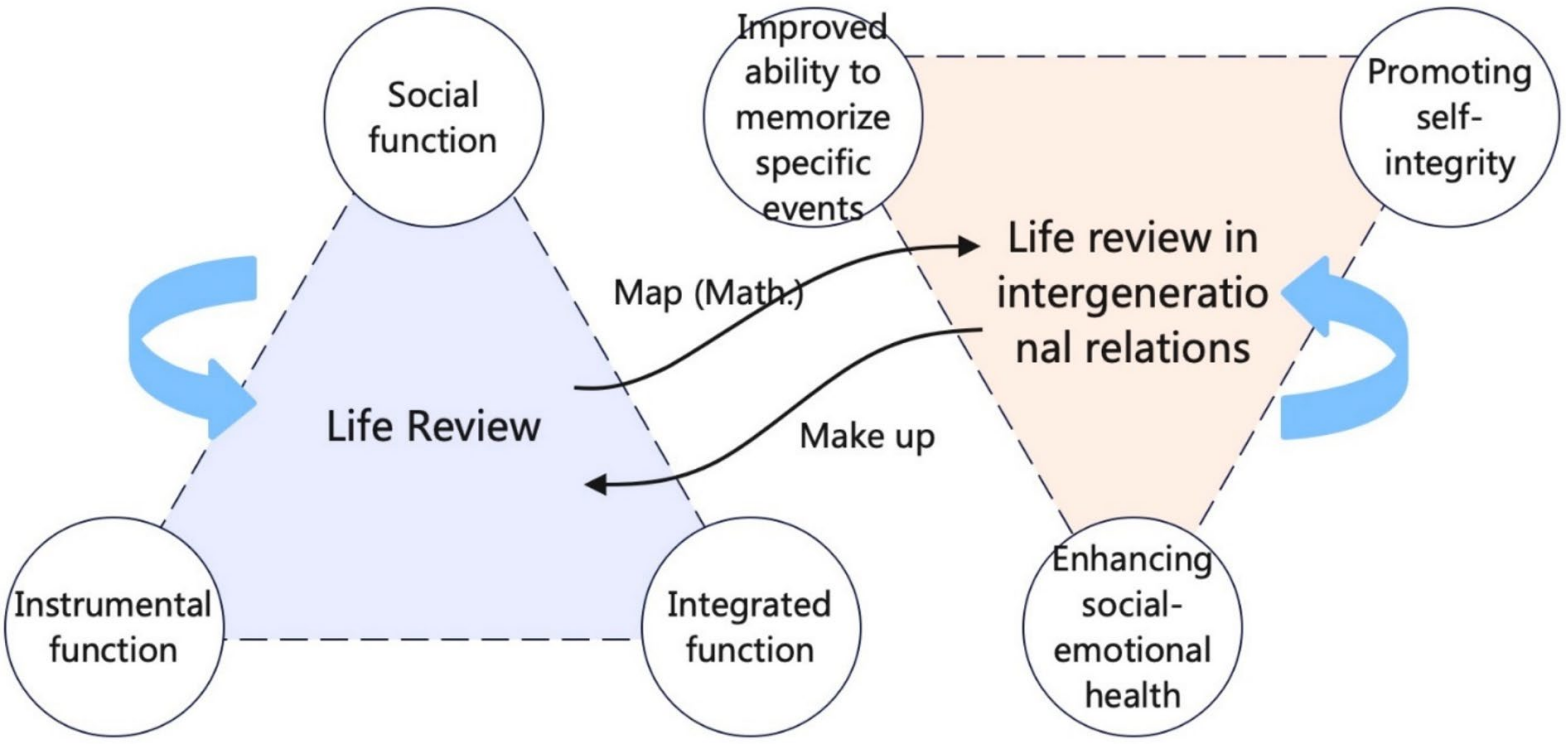
Life review in the intergenerational context.

Current literature extensively explores life review methodologies and intergenerational play as separate domains. Life review is mainly achieved through recollection, different fields define reminiscence, with the cognitive field of G Nigro (5) defining it as the widely recognized perspective of re-experiencing the past, either individually or in groups (6, 7). Reminiscence is a passive and spontaneous process that may be part of a life review, but is not synonymous with it. In a life review, people chronologically organize their life events to ensure a more structured theme (8), and it is a structured and systematic process consciously implemented with voluntary participants (9). The three functional categories of a life review (Figure 1) were (8, 10) (1) a social function that enhances connections by sharing private memories with others; (2) an instrumental function that allows for the recall of past experiences and helps people face current difficulties; and (3) integrative function that creates identity by connecting with the future. Life reviews have also been shown to improve the ability of people with dementia to remember specific events, encourage communication and socialization, and enhance the socioemotional wellbeing of older adults by improving intergenerational support to reduce depression and loneliness (11). Furthermore, the role of listeners is particularly prominent in facilitating successful life reviews, as they can identify the characteristics of narrators and apply effective strategies accordingly. Integrating new pathways within intergenerational interventions can significantly enhance communication between listeners and narrators, improve the quality of their relationships, and foster shared enjoyment of the process. Life review cultivates self-integrity—a sense of coherence essential for reconciling life trajectories (12)—while empowering older adults as storytellers and younger generations as recipients of cultural heritage (8).

Existing life review interventions apply technology and digital systems. For example, photos, videos, and music have introduced multimedia to collect popularity data based on comments on photos and music to facilitate recall (13). Several advantages of applying digital technologies to help older adults with life reviews to improve their self-esteem and life satisfaction have been suggested (14, 15). First, digital technologies can enable greater engagement by alleviating motor and sensory impairments and compensating for memory deficits. Second, digital technologies can enable teletherapy and customization to reduce the burden of providing therapy by collecting and organizing basic personal materials. Finally, digital technology can be used to assess participants’ progress and system use. Many older adults attempt to connect to today’s society through digital technology, but not all are adept at using digital technology to stay connected to their family groups, and their mobility is significantly limited (16).

Digital games (17, 18) have been used to promote reminiscence as a part of attempts at digital technology. The interactive nature of the game distinguishes it from other passive media, such as movies, and can actively promote life reflection for participants (19). This interactivity allows participants to easily sympathize with the game characters and relate their lives to the stories in the game. Several studies have used games for recall. The Memoir Monopoly game uses an iPad to integrate participants’ materials into the game so that older adults can recall memories based on in-game photos, music, and quizzes (20). The game “What Remains?” employs a persuasive game design that allows individuals with Alzheimer’s disease to tell stories to their caregivers based on pictures of their personal belongings (21). Therefore, gameplay serves as a key intervention strategy for enhancing life review. It can support participants in achieving better learning outcomes through motivation and interaction (22, 23). Older people have a certain familiarity with the game itself (24), as many have been exposed to different forms of gaming from a young age. Research has shown that digital games are associated with the wellbeing of older players and can provide cognitive and physical benefits (25).

Playing together increases the enjoyment of the game and the positive emotions of players. These benefits are particularly enhanced when older and younger generations play together (26). Younger people playing games with older family members (e.g., parents, grandparents, and uncles/aunts) can be effective at reaping the benefits of fun and bonding with family members (27). Intergenerational games help strengthen family bonds, enhance reciprocal learning, increase the understanding of other generations, and reduce social anxiety (28). In addition, intergenerational play fosters positive perceptions of members of other age groups, increases the breadth and depth of self-disclosure, and promotes relational intimacy between younger and older adults (29). However, gaps in the technological experience and skills between older and younger people can lead to unbalanced interactions. When playing intergenerational games, younger people play the role of teaching the game’s operations, and as a result, communication between older and younger people is not symmetrical and reciprocal (27). Often, during gameplay, older adults adapt to and understand game activities through the guidance of their younger partners. Older adults asked questions and received feedback from their younger partners to learn about game mechanics. Younger partners responded to the problems they had, corrected errors, and explained game rules and strategies. The main challenge in intergenerational play for younger partners was the unfamiliarity of older family members with the rules and mechanics of the game. Skill gaps, knowledge of different cohorts, and physical and cognitive decline in older adults may be barriers to intergenerational play. Therefore, more research is needed to enhance reciprocal interactions between two generations in families during play.

However, few studies bridge these areas to address how games can enhance life review within families. While digital technologies, such as multimedia reminiscence systems, have shown promise in supporting older adults’ self-reflection (14), their focus on individual rather than collaborative engagement limits their potential to foster bidirectional dialogue. Similarly, existing intergenerational games often prioritize entertainment over structured reflection, failing to integrate life review’s therapeutic benefits. Although games such as *Memoir Monopoly* demonstrate the viability of digital platforms for memory recall (30), they primarily target clinical populations or caregiver relationships, overlooking the unique dynamics of familial bonds. Moreover, many interventions inadvertently reinforce skill imbalances, with younger participants dominating technical aspects rather than fostering equitable exchanges of wisdom and experience (31). Such efforts could align with public health priorities by mitigating social isolation and promoting mental wellbeing across generations, yet remain underexplored in both academic research and practical design frameworks.

To address these limitations, metaphorical life games have been proposed as a potential solution. By embedding universal life themes into gameplay, such games can create shared narratives that transcend generational divides, offering a neutral platform for storytelling and reflection. This study aims to investigate the role of metaphorical life games in enhancing intergenerational communication within families, addressing the underexplored intersection of gamification and collaborative life review. This study employs “*The Story of a Life*,” a digital game depicting universal life stages (e.g., birth, education, and aging), to explore how metaphorical gameplay facilitates intergenerational communication. The game’s simplicity, flexible objectives, and thematic stages make it suitable for life review activities. Specifically, we seek to answer two research questions:

*RQ1:* Is the metaphorical game effective in promoting mutual perceived closeness between older and younger adults in families?

*RQ2:* What mechanisms underlie the effectiveness of life metaphor games in enhancing intergenerational family life review?

In this study, we propose intergenerational games for life metaphorical games as alternative settings to promote rich dichotomous communication for life review and to create an enjoyable atmosphere between older generation speakers and younger generation listeners through game experiments with semi-structured interviews with older and younger people from the same family under a life review theme experiment with test data. Proponents argue that metaphorical elements reduce the pressure of direct reminiscence, allowing participants to project personal experiences onto abstract scenarios, thereby lowering emotional barriers. Critics, however, caution that overly simplistic or culturally mismatched metaphors may fail to resonate with diverse lived experiences, while asymmetrical gameplay mechanics could perpetuate existing communication imbalances. Despite these challenges, preliminary studies suggest that well-designed metaphorical games can harmonize structural guidance with spontaneous interaction, balancing therapeutic intent with playful engagement. By analyzing gameplay interactions and thematic dialogue, this research contributes actionable insights for designing inclusive, culturally adaptive games that empower both generations to share, reflect, and connect.

## 2 Research methodology

### 2.1 Research setting

**Figure 2:**
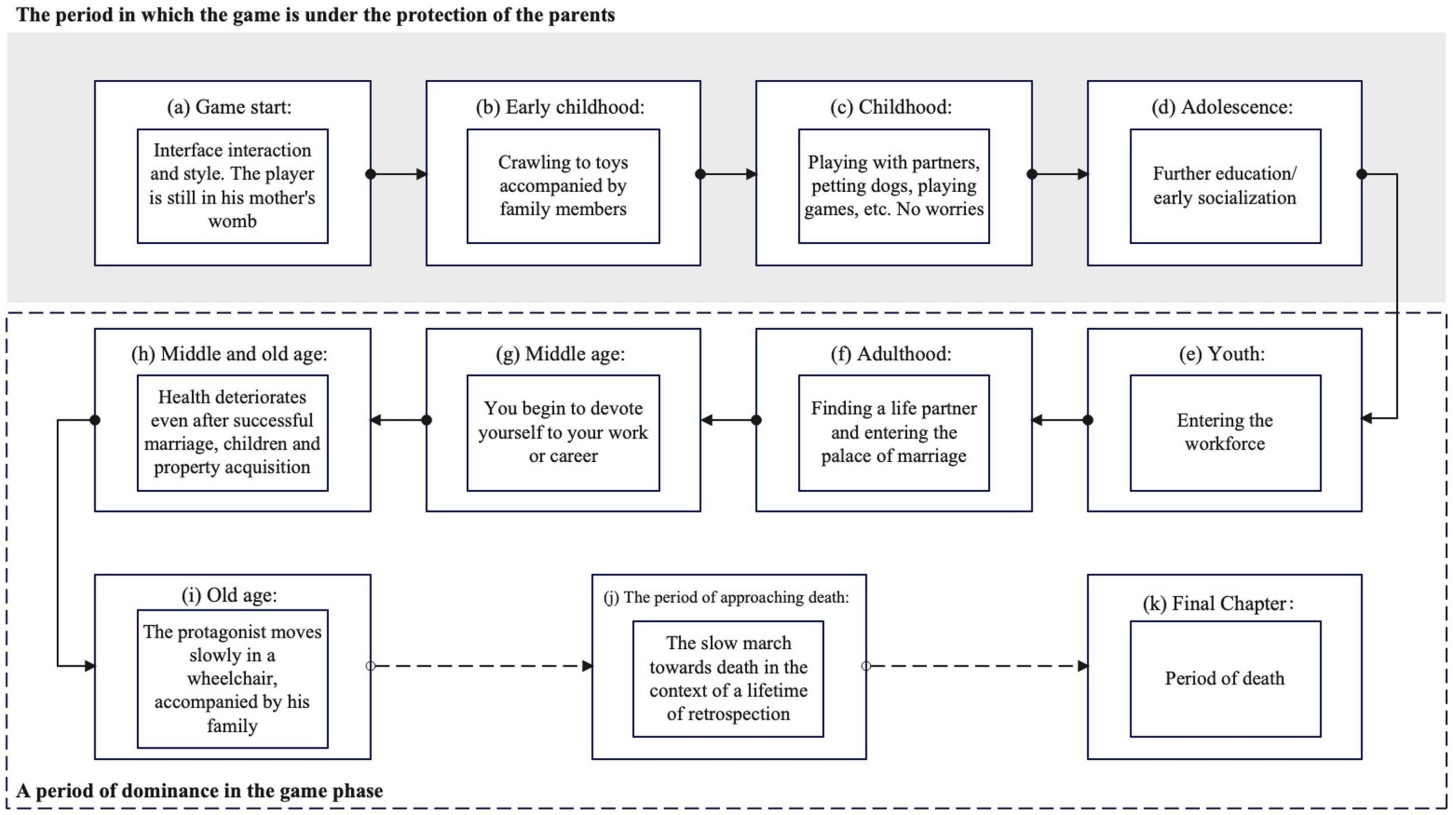
Different life themes in the game.

Our research was conducted using the game *The Story of a Life* as the platform. This game depicts an individual’s life journey from birth to death. Several aspects of the game are relevant to this study (see Figure 2). First, it supports reminiscence, as it encompasses the human lifespan (birth, aging, illness, and death), encapsulating life events that encourage players to reflect on their experiences. Second, the game’s interface elements and controls were relatively simple. Given that complexity may disrupt intergenerational communication during life reviews, the characters in this game only need to follow normal movement patterns (moving up, down, left, and right, along with jumping), making it suitable for our research context. Finally, the game is flexible and informal and lacks strict operational constraints and specific objectives. During gameplay, older participants (SP) and younger participants (YP) can pause their actions at any time to converse, facilitating the capture of rich interactive practices between the two age groups.

### 2.2 Experimental design

More contextualized research on the process of game design can aid user experience stickiness (32). Therefore, game design is based on a dichotomous approach to an experimental design for user needs research and contextual analysis. Because we chose games that can be easily learned by novice players, previous gaming experience and technical skills in using digital devices were not considered hard criteria for screening. A total of 24 pairs of participants were included in this study (Table 1) to ensure the accuracy of the data and comparability of the experiment, as the user group consisted of older adults and their grandchildren who had been exposed to the game in a psychologically healthy (i.e., no history of gaming addiction or adverse psychological effects) manner. All participants were provided with information about the study and agreed to participate before starting the activities. We debriefed each activity. Participants consistently reported that the activities were positive experiences and facilitated their interaction with each other.

**Table 1:** Participant demographics.

Group	SP	YP
Age	61–65 (4), 66–70(12), 71–75 (6), 76–80, (2)	18–22 (16), 22–26 (8)
Gender	Male (10), female (14)	Male (13), Female (11)
Relationship	Grandparents to grandchildren (19), maternal grandparents to grandchildren (5)	
Educational background	Primary/junior high (15), senior high (4), undergraduate (4), graduate (1)	Primary/junior high (0), senior high (1), undergraduate (15), graduate (8)
Family income (monthly)	Low: below 8,000 RMB (6), medium: 8,000 to 20,000 RMB (16), high: above 20,000 RMB (2)	

The experimental design comprised four steps: a pre-game questionnaire, joint gameplay, paired discussions, and a post-game questionnaire.

1. Step 1 (Questionnaire Q1): Prior to gameplay, participants were asked to rate their perceived closeness to one another on a 7-point scale (1 = completely disconnected, 7 = very close).
2. Step 2 (Joint Gameplay): Young participants were instructed to pose questions at different stages to encourage older participants to describe a particularly memorable experience related to various life themes. Each pair of participants typically experienced 5–6 life themes (out of a total of 9), spending an average of 30–35 min playing together. The younger partners assisted the older participants in navigating the game. When a pair encountered difficulties, researchers provided support but aimed to minimize their involvement.
3. Step 3 (In-depth Interviews): Following gameplay, participants engaged in a discussion about their memories and feelings regarding each game scene for 20–25 min. Young participants filled out a form distributed by researchers, detailing how the older participants reflected on each stage of the game. Each row on the form corresponded to a different life stage, with an adjacent blank column for the younger participants to summarize and record the older participants’ recollections. This was followed by further discussions on the game’s strengths, weaknesses, and potential improvements.
4. Step 4 (Questionnaire Q2): After the in-depth interviews, participants rated their perceived closeness to one another again on a 7-point scale and provided explanations for any changes in their ratings.

### 2.3 Data analysis

Post-game interviews were conducted by two researchers not involved in gameplay observation. Participants (SP and YP) were interviewed together in a private room, with researchers posing open-ended questions about their experience. A third researcher observed and recorded natural interactions during gameplay but did not intervene. Written informed consent to participate in this study was provided by the participants.

First, we analyzed and recorded the pre- and post-arithmetic mean (AM), standard deviation (SD), and reasons for the 24 perceived closeness ratings (RQ1). The transcript data were qualitatively analyzed using NVivo, based on all recorded conversations between SP and YP during the event (Approximately 12 h of recorded conversations during gameplay and interviews were analyzed). After multiple readings of the transcript results (step 1), the first author coded the entire dataset following reflexive thematic analysis (RTA) as outlined by Braun and Clarke (33). Meaningful units were extracted and coded to summarize their content. The codes were then merged to form the subthemes and overarching themes. In the first round of merging, 3 themes and 18 subthemes were created. (Steps 2 and 3). The three authors reviewed and defined the themes through discussion. After returning to the dataset and examining the codes and themes several times, the final number of subthemes was reduced to 10 with three broader overarching themes. (Steps 4 and 5). The reliability of the analysis was ensured by analyzing conferences throughout the analysis process: three conferences involving two co-authors and one conference involving three co-authors. During the conference program, the first author provided a walkthrough for analysis in its current state. Following this, the content of each preliminary topic was presented and discussed sequentially. Disagreements and uncertainties were resolved through discussion. (Step 6). Finally, we expanded the analysis to include game elements that contributed to these themes. The basic elements of the game (except for the player) were explored in three categories: structure, metaphor, and interaction (34), and the link between communication themes and suggestions for game design was made through participant evaluations and recommendations (RQ2).

This study was a qualitative exploration. Nonetheless, we found it valuable to provide the reader with a sense of the prevalence of different themes and sub-themes among the participants. To this end, we will use the following terms to denote the number of participants in each group associated with the results shown in the results section: a few (1–4 participants), some (5–12), most (13–19), and almost all (21–24).

## 3 Results

### 3.1 Results of questionnaire analysis

An analysis of the data from the perceived closeness questionnaire between the older and the corresponding younger groups (Table 2) showed that in the younger group, the AM values in Q2 (SD = 0.974, AM = 4.083) were significantly higher than those in Q1 (SD = 0.897, AM = 5.750), and the majority of the participants in the younger group changed their perceptions of their elders considerably, while a few of the participants remained unchanged. In the older group, there was also a further increase in AM values in Q2 (SD = 0.761, AM = 5.246) compared with Q1 (SD = 0.711, AM = 6.283), with almost all participants in the older group benefiting from this. In addition, we found that in each intergenerational pair, older adults’ perceived closeness to younger adults was higher than younger adults’ perceived closeness to them both before and after the activity. Thus, the Life Metaphor Game can be effective in promoting mutual perceived closeness between older adults and young adults during life review activities. Based on this, we further explore gamification design strategies for using life metaphor games to effectively promote intergenerational reviews.

**Table 2:** Average ratings of perceived closeness (R1, R2).

Number	YP		SP	
	Q1	Q2	Q1	Q2
1	3	5	5	7
2	4	6	6	7
3	3	3	5	6
…	…	…	…	…
22	5	6	6	7
23	3	5	4	6
24	4	6	4	5
SD	0.974	0.897	0.761	0.711
AM	4.083	5.750	5.246	6.283

### 3.2 Results of the thematic analysis

The six themes that emerged the most during this exercise were identified were: (1) reminiscing, (2) looking forward to the future, (3) teaching life lessons, (4) communication as a life experience, (5) explanation of game mechanics, and (6) metaphorical expression of the game. Table 3 presents the definitions and sources for each theme. The sources included the people who participated in the event and the connection between these themes and the metaphorical game. The theme identified only from the older participants’ expressions was “memories and the transmission of life experiences.” The theme identified only from younger partners was “the teaching of game mechanics.” In addition, from both groups, the “exchange of perspectives on the future, recent life situations, and the explanation of the metaphorical expressions of the game” were identified.

**Table 3:** Life review themes in intergenerational families.

Topic	Definition	Source	
		Crowd	Metaphorical game elements
Reminiscing	Re-experiencing Perspectives from the Past	SP	Structural elements Interactive elements
Looking forward to the future	Perspectives on development and planning, death	SP, YP	Structural elements
Teaching life lessons	Individuals passing on knowledge, skills, and coping strategies that they have accumulated in their own lives to other individuals	SP	Structural elements Metaphorical Elements Interactive elements
Communication as a life experience	Sharing of information among individuals about their recent life status, experiences, feelings, and changes	SP, YP	Metaphorical Elements
Explanation of game mechanics	Analyzing and guiding the core elements of the game, such as rules, operating procedures, and strategy skills	YP	Interactive elements
Metaphorical expression of the game	Explaining the meaning of images, elements, or objects in the game.	SP, YP	Metaphorical Elements

### 3.3 Metaphorical game elements and intergenerational communication

#### 3.3.1 Structural elements: game scenarios

The game “*The Story of a Life*” consists of 11 stages, each of which is judged by the scene in which the characters are placed, based on the structural elements of the game, such as the movement of the characters in the background, the houses, and the tombstones, created natural prompts for intergenerational dialogue. For instance, during the “marriage” stage, YP6 (20, female) pointed to the wedding scene and asked her grandmother (SP6, 73): “Do you see those characters in suits and wedding dresses? How did you and Grandpa get together?” SP6 paused, smiled, and replied: “It reminds me of our wedding day—no fancy dresses, just a simple ceremony. But we held hands tightly, just like those characters.” In relation to the structural elements of the game, the life review themes of “reminiscing,” “looking forward to the future,” and “teaching life lessons” were frequently addressed. The majority of older participants were prompted by the younger participants to realize that the next stage of life was entering and discovering their memories, as the game itself did not have clear indications of stage transitions.

As some participant groups progressed to the middle and late stages of the game, events that were not experienced by younger or older participants facilitated communication between them. For example, the majority of the younger participants, when they saw their characters being manipulated out of school and in the workplace, asked the older participants how they felt when they were there and expressed their opinions. As the pair advanced to the “workplace” stage, YP3 (23, female) observed her character entering an office and turned to SP3 (76, male): “Grandpa, what was your first job like?” SP3 leaned back, recalling: “I worked in a factory. We had no computers—just tools and teamwork. But those days taught me resilience.” SP3 (23, female) replied: “I plan to go back to develop after I graduate, I do not want my life to be too tiring and I do not think being rich makes me happy. I prefer to be with my friends ……” Additionally, some of the older participants were saddened to see their characters as “tombstones” because they were not healthy, and although they had not experienced death, they told stories and talked about their futures based on their imaginations. For example, the “tombstone” scene prompted SP21 (78, male) to reflect on mortality: “Seeing this… I worry about being a burden.” His grandson (YP21, 22) quickly countered: “You’ve never been a burden. Your stories help me understand our family’s history.” “We defined this information as the transmission of life experience” “vision about the future.” These exchanges highlighted how structural transitions bridged past and present, allowing older participants to contextualize their experiences within the game’s narrative.

#### 3.3.2 Metaphorical elements: play space

The game depicts a person’s life through a lifetime of universal experiences that form the main thread of the game (the main direction of the game’s development), with each stage having a different ambiance in the game space, which includes metaphorical objects for the player’s interpretative pleasure. In relation to the metaphorical elements of the game, the theme of “metaphorical expression of the game” often appeared in relation to the game space and objects. Younger participants were primarily responsible for interpreting the meanings of metaphors, but older participants also interpreted their own meanings. Older participants empathized with the expressions and applied metaphors to their lives, thus creating ‘memories.’ For example, the clock-like object in the background of the game was always accompanied by changes in the game’s progression, becoming smaller and turning clockwise as the player controlled the character throughout his or her life, sparking interpretive discussions. Guided by young participants, when SP9 (65, male) noticed the clock shrinking as gameplay progressed, he mused: “Yes, I felt it. The space is slowly changing as the character moves, the hands and size of the clock in the background… This could indicate our progress in the game. Just like our life, although it’s not this clear-cut like the game, not knowing which moment we’ll face death, we should be positive about the present moment, only so that when you get to my age it’s fulfilling to look back on it, instead of feeling idle about your past, cherish the present. Even if my time is shorter, I want to make it count.” These interactions revealed how metaphors allowed participants to project personal meanings onto abstract symbols, fostering reciprocal emotional exchanges.

The theme of “communication as a life experience” occurred at almost any stage of the activity, as the different scenarios in the game were exactly what the older or younger participants were experiencing. In addition, several objects accurately evoked the sharing by older participants or provided clues for younger participants to ask appropriate questions. These are related to reasons for participation, time spent with friends, progress in learning, and physical condition.

In addition, the game featured water, a clock, and melodies. Some participants provided positive comments about the sounds and color schemes. However, there were still a small number of participants who felt that the color presentation and music in the game were a bit too monotonous. SP6 (77, female) mentioned: “The color scheme of this game is simple.” SP6 (21, female) replied: “The music of the game made it more soothing for me to listen to, but the colors were a bit too simple for me.”

#### 3.3.3 Interactive elements: game manipulation

Despite the simplicity of the game controls, older participants were still unable to perform the actions required in some specific scenarios. For example, during the “old age” stage, SP16 (68, male) struggled to navigate a layered plane: “How do I climb up? There’s no ladder!” His grandson (YP16, 19) guided him: “Just move left—the game lets you jump sideways here.” SP16 chuckled: “Ah, games today are clever! In my time, ladders were real, not pixels.” YP16 grinned: “But you are learning fast!” This dynamic extended to metaphorical interpretations. The majority of older participants were afraid to take control of their characters and move forward when faced with water. Older participants often sought guidance and noted that they did not relate to the game or could not determine how to play it alone. Recalling without the guidance of a younger partner was also a challenge for older participants, who explained the controls of the game and the meaning of the objects. Many patterns of communication were identified, as older participants asked how the controls were, and younger participants explained them to them. We refer to this theme as the “explanation of game mechanics.”

At the end when the characters were climbing upward and all the previously experienced scenes appeared in the background of the game, the communication theme started with the younger partner’s explanation of the game mechanics, followed by the older participants’ “metaphorical interpretation” and “reminiscence.” Depending on the interpretation of this action, different themes of recollection followed. Some participants expressed it as a reminiscence before the person’s death. SP1 (70, male) mentioned: “Take a quick look, the game character has been climbing upwards uncontrollably with the ladder, why is that?” SP1 (21, male) replied: “It’s okay, it’s supposed to be a game mechanic, you see in the background of the game there is a constant appearance of what the character just went through, now it might be similar to the form of a movie playback” SP1 (70, male) also said: “Oh I see, has it come to the end of its life? I have seen similar dramas where a person would re-experience life’s experiences in their mind when they are about to die, like now.”

Some other older participants seemed to have a deeper understanding. They made a connection between the phenomenon and real life, looking back on their own lives and giving some life lessons to the younger participants. For instance, SP14 (male, 76) reflected, “The game made me realize how solidarity shaped our lives,” while YP3 (female, 23) shared, “Hearing Grandpa’s stories helped me appreciate his resilience.” These narratives underscore how symmetrical interactions fostered mutual understanding. A few older participants also rated the game’s portrayal of old age as brief, simplistic, and frustrating. They suggested that a thorough understanding of older adults’ lives is necessary to provide an ideal life review game that identifies and integrates factors that help players convey more positive emotions, such as comfort, joy, and action. Additionally, a few older participants noted that the imagery in this game is based on Western culture and suggested a design that is more in line with the player’s cultural background.

## 4 Discussion

This paper presents the results of a two-stage survey in which 24 pairs of older and younger people in families were selected to participate in activities. A questionnaire study of the impact of games on perceived intergenerational closeness, as well as an interview study of design strategies for games to facilitate life review in intergenerational communication, were conducted. According to the interviewees, the central dynamic that allows metaphorical games to facilitate intergenerational communication can be defined as creating and building links between the three dimensions of structure, interaction, and metaphor. In general, the interviewees described their experience of the activity, emphasizing the stimulating nature of the game elements for communication throughout the gaming experience, as well as the de facto need to cope with the sources of change in the gaming environment. One interviewee (SP19, male, 73) noted the duality of gameplay as both a structured activity and a spontaneous bonding opportunity: ‘The game gave us a framework, but our conversations flowed naturally, possibly due to possessing blood ties themselves.

Based on the findings we presented, we discussed the proposed research questions. Following this, we pointed out the limitations of the study as well as avenues for future research.

### 4.1 The feasibility of metaphorical games in intergenerational family relationships

Existing research has found effective results regarding games that can be effective in communication (35), but has not explored their feasibility in family intergenerational specific contexts. In response to RQ1, we found that living metaphor games within intra-family relationships can also enhance their mutual perceived closeness and be a useful tool for facilitating a variety of communication topics during intergenerational life review activities. Both younger and older players were allowed to play a variety of different role expressions. Second, the effectiveness of this game also has a relationship with older adults’ self-disclosure and the intimacy between family generations themselves. This is because we found a few results in the data of some participants that were not favorable, and their perceived closeness to each other through this intergenerational activity did not produce a large change.

### 4.2 Mechanisms by which life review promotes intergenerational communication

For aging and public health, enhancing the interaction and communication between older adults and young people is an important way to achieve their happiness and wellbeing, and thus their health, and the specific area of this paper is this intergenerational communication. Gamification helps older and younger people develop a cooperative relationship in which goals are accomplished with help and reciprocity. The relationship is more egalitarian, with participants valuing and respecting each other’s skills. Such egalitarian interactions align with public health priorities to mitigate social isolation and promote mental wellbeing among aging populations (1, 11). This can be described as flourishing, which is a unique aspect of happiness as defined by Killen and Macaskill (36) as “supportive and beneficial relationships that contribute to the well-being of others and are respected by others”. Although this study did not measure the happiness of the participants, young and old alike found the activity to be very beneficial and likely to improve their wellbeing. This is a positive intergenerational activity, and it is hoped that some of the lessons learned from this study can be utilized in the design of future technologies designed to support a variety of intergenerational strong ties.

**Figure 3:**
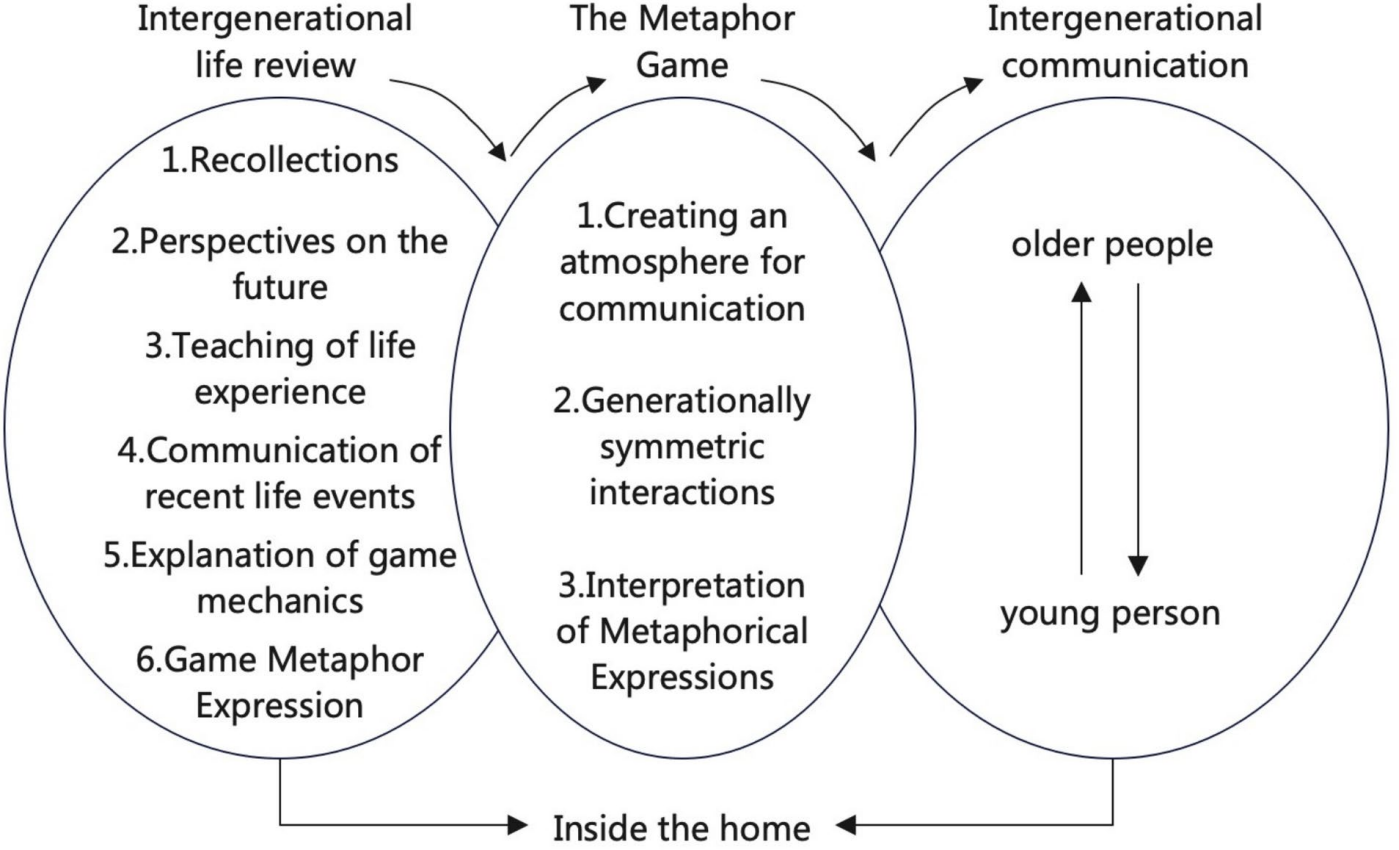
Mechanisms of metaphorical game-based life review for intergenerational communication.

Based on our findings, we developed a strategic mechanism for life review to promote intergenerational communication. This mechanism, structured as a model comprising ‘communication atmosphere creation,’ ‘symmetrical intergenerational interaction,’ and ‘metaphorical expression interpretation,’ emphasizes the process model found in the daily practice of families through the metaphorical game design of life review (Figure 3). Respondents emphasized reciprocal relationships and overlapping dimensions of the game design process. The specific mechanics of our findings and recommendations for game design are discussed in the following sections.

#### 4.2.1 Creating an atmosphere for communication

Our findings in this study suggest that games provide an opportunity for both participants to comfortably communicate and review their lives, addressing public health concerns around the loneliness of older adults and youth disconnection from their familial heritage. The majority of the participants in both groups in the gaming workshops responded positively to the process as they were able to enjoy the experience through games involving text, video, and music rather than one-to-one interviews in a closed room—echo findings from public health interventions using digital tools to strengthen social cohesion (14, 18). People also generally found playing games to be a fun activity. The results of this activity support Fan Zhang’s (37) findings that intergenerational gaming promotes interactions between two generations and fosters positive intergenerational perceptions. This applies to family relationships as well. Younger partners can use metaphors to ask appropriate questions during the game, and through questions related to the game, older participants have the opportunity to objectively review their lives.

Furthermore, conversational themes were naturally renewed at each stage, as gameplay took precedence over storytelling. Participants in this study similarly criticized Story of *The Story of a Life* because it offered only two-story paths, and it was not rich in color and musical variation. Games designed to support life review should reflect a variety of real-life experiences rather than stereotypical lifestyles. Although this is an activity within the family intergenerationally, older participants have a low desire for representation when they have low openness and couple intimacy, and professional therapy may be better able to help those who refuse to recall past events or have experienced trauma than intergenerational life review activities.

Therefore, future designs should prioritize accessibility features (e.g., adjustable difficulty and culturally tailored narratives) to align with public health goals of inclusivity and equitable aging support (38). Providing the opportunity to reflect on the player’s life in an integrated way, the player can choose the game story that best suits his or her life experience and have the colors and music of the scene to match. To do this, metaphorical game content that promotes intergenerational communication must convey rich stories that recall challenges, difficulties, and happy moments, while balancing each stage with more complex and sophisticated storytelling. Games provide enjoyable emotional outcomes, personal growth, and perceived benefits for older adults (39). From a lifespan perspective, meaning in digital games comes from players fostering connections, nurturing self and others, and contributing to society (40). Although life review games are not primarily focused on fun, successful life review games support personal growth and foster healthy relationships.

#### 4.2.2 Generationally symmetrical interactions

We identified the possibility of engaging in dialogic balancing during the course of the activity, the reciprocal learning observed, where older adults share life wisdom and younger participants teach digital literacy, mirrors public health strategies that leverage intergenerational partnerships to combat ageism and technological exclusion (4, 28). Such symmetry counters the “digital divide,” often isolating older adults (16), while empowering youth as agents of familial health literacy. For example, older participants’ critiques of stereotypical aging portrayals in games highlight the need for design frameworks reflecting diverse aging experiences, a priority in public health advocacy (11, 41). Research on intergenerational learning also emphasizes that social interaction should not be a one-way process, but should benefit both generations (4). Previous research on intergenerational play has emphasized the imbalance in interaction (37). This is because younger people are more skilled at using digital games, whereas older people usually ask questions; younger people always play a role in teaching older participants how to use the game. However, in game-based life review activities, more reciprocal relationships can be established as older adults can positively convey cultural values and wisdom from their life experiences.

In addition, the study confirmed that respondents viewed flexibility as an important aspect of game design (42), which is reflected in the effectiveness of the direct link between games and life. This is because it is necessary to cope with change and ensure the potential adaptability of the game process. The use of digital games for life review activities allows for the addition of game-related dialogue to stories about memories, such as metaphorical explanations, explanations of control methods, and appreciation and evaluation. The need to adapt game design methods should also be emphasized, emphasizing the contextual nature of variability embedded in fixed practices and their effectiveness (the role of change). Such adaptability is seen not only as a response to change but also as an essential attribute in enhancing the responsiveness of programs to a wide range of stimuli and influences, inspiring older and younger generations, and fostering family cohesion. In this context, one might distinguish between two types of variables: “variables related to the stimuli presented to participants (e.g., novelty and number of stimuli)” and “variables related to the design process (e.g., participant characteristics, group size, and time available)” (41). Thematic constraints can also have a positive impact on the game design process as they promote focus, faster decision-making, and ultimately contribute to the creation of innovative games. As the words of the interviewees seem to confirm, designers usually do not see constraints as limitations, but rather as catalysts that motivate them.

Therefore, old age should be expressed more richly and positively in metaphorical games that promote life review. By embedding adaptive mechanics (e.g., customizable life stages), games can emulate public health models that prioritize individualized aging trajectories (25). Some older participants were dissatisfied with scenes of old age because they were expressed as brief, simple, and frustrating. Ordinary life in old age should not be described in terms that are too static, passive, negative, or pessimistic. Designers must have a deep understanding of aging in order to create rich narratives. Careful consideration of minority groups is also necessary to help players better understand metaphors that are similar to their own cultural backgrounds. In addition, new technologies are often developed based on simple assumptions about older people. They present a more complex reality later in life based on older people’s perspectives (38). Designers should allow older adults to participate in game design workshops, which may help to identify ideal approaches and reflect immediate feedback. Collaborative gameplay also aligns with the WHO’s “Decade of Healthy Aging” goals, emphasizing intergenerational solidarity as a determinant of societal wellbeing (1).

#### 4.2.3 Interpretation of metaphorical expressions

Metaphors serve as conduits for translating personal narratives into collective health dialogues. Younger participants’ role in decoding game metaphors parallels public health interventions, training youth as caregivers or “memory facilitators” (12). This dynamic reinforces family resilience, a key factor in buffering against age-related stressors (6). Throughout the intergenerational activities, it is evident that the role of the young partner is crucial in family life. People usually talk about the game itself during intergenerational play; the conversation can naturally shift to small talk and discussion of important life issues (26). For instance, discussions triggered by tombstone imagery (symbolizing mortality) allowed older adults to articulate end-of-life values, a process linked to improved psychological preparedness in aging populations (8). Reviewing the activities in this study, they (YP) asked questions to elicit memories, explained the metaphors in the game, and directed the playing of the game. Despite the relative simplicity of the game controls, older participants mentioned that it was difficult for them to enjoy the game without the help of younger people. Emotional interactions between commentators and listeners play an important role in recall (12). Younger participants could use a variety of communication strategies to successfully trigger reminiscence in older participants, but there were differences in individual abilities as facilitators. Successful life review activities require pre-session training for younger partners to train in game methods and effective communication strategies.

Specifically, metaphors are critical for game designers and participants. Given the sustainable exploration or mining characteristics of game mechanisms, it is difficult to measure the success of a game through design documentation alone. On the one hand, the metaphorical design process transforms game designers into active players, enabling them to critically assess game performance and identify areas that may hinder player engagement. On the other hand, it can help players to motivate their gameplay and help them discover metaphorical elements to establish communication paths during the metaphorical game experience, where, as the interviewees reported, a singular vision may indeed fail to stimulate communication instincts and lead to a loss of perspective on the viability of the overall project.

Thus, future games could integrate evidence-based metaphors (e.g., seasonal cycles for life transitions) validated in gerontology research (7) to amplify therapeutic outcomes. Recollection is effectively facilitated when participants understand and empathize with the meanings of the metaphors in the game. Metaphorical games that promote intergenerational life recall should link to real life, and setting up a game design that is relevant to real life will help promote intergenerational life recall. Recall was well facilitated when older participants understood and empathized with the metaphorical meaning of the game. Because the metaphorical game of life, Story of a Lifetime, provides a metaphorical element, it can encourage conversations related to personal stories. Intergenerational play can promote self-disclosure and relational intimacy. By talking about the game itself during intergenerational play, the conversation can naturally shift to small talk and discussion of important life issues. Public health practitioners might adopt such gamified approaches to scale community-based reminiscence therapies, addressing systemic gaps in elder mental health support (14).

## 5 Conclusion

This paper has limitations, even if it has useful theoretical and practical consequences. First, while the game clearly illustrates the specific performance of improving life review through gaming, other games may use different mechanisms involving different audiences. For this reason, we encourage follow-up studies to investigate the performance of life review in various cultures, among individuals with varying levels of game familiarity, and on various gaming platforms.

Second, this study was intended to propose the relationship between metaphorical game elements and life review and its mechanisms, but it did not systematically evaluate and validate them, so follow-up studies can further assess the validity of this period.

Finally, the study was intended to explore the mechanisms of metaphorical games that promote intergenerational communication, rather than a whole theory, and follow-up studies can further enrich the theoretical model of gamified intergenerational communication proposed in this study. Despite these limitations, the study experimented with a new family activity by combining intergenerational games and life reviews and showed that these activities can be effective in promoting successful aging and public health.

Although games are primarily designed for young people, many middle-aged and older players have been playing them since childhood. The demand for games from these players has led to an established gaming industry. Successful life review games leave a rich impression on players through memories associated with the game’s story. In addition, they can help older adults take on the role of storytellers, enabling them to continue to contribute to their communities and maintain their social status. By fostering intergenerational cohesion and reducing older adults’ isolation, these findings align with public health goals of promoting mental wellbeing across generations. Future game designs targeting aging populations could integrate public health frameworks to amplify societal impact.

## Data Availability

The original contributions presented in the study are included in the article/supplementary material, further inquiries can be directed to the corresponding author.

## References

[1] Bengtson VL. Beyond the nuclear family: the increasing importance of multigenerational bonds: the burgess award lecture. J Marriage Fam. (2001) 63:1–16. doi: 10.1111/j.1741-3737.2001.00001.x

[2] Hooyman NR. Social gerontology: A multidisciplinary perspective. MA: Pearson (2008).

[3] Reis L Mercer K Boger J. Technologies for fostering intergenerational connectivity and relationships: scoping review and emergent concepts. Technol Soc. (2021) 64:101494. doi: 10.1016/j.techsoc.2020.101494

[4] Ropes D. Intergenerational learning in organizations. Euro J Train Develop. (2013) 8:713–27. doi: 10.1108/EJTD-11-2012-0081

[5] Nigro G Neisser U. Point of view in personal memories. Cogn Psychol. (1983) 15:467–82. doi: 10.1016/0010-0285(83)90016-6

[6] Gedi N Elam Y. Collective memory—what is it? Hist Mem. (1996) 8:30–50.

[7] Butler RN. Successful aging and the role of the life review. J Am Geriatric Soc. (1974) 12:529–35. 10.1111/j.1532-5415.1974.tb04823.x 4420325

[8] Haber D. Life review: implementation, theory, research, and therapy. Int J Aging Hum Dev. (2006) 63:153–71. doi: 10.2190/DA9G-RHK5-N9JP-T6CC, PMID: 17137032

[9] Wang C Liu Z Chen T Wang J Zhang X Han B. Intergenerational support and depressive symptoms in old age: the difference between urban and rural China. Front Public Health. (2022) 10:1007408. doi: 10.3389/fpubh.2022.1007408, PMID: 36466487 PMC9709321

[10] Clements WM. Reminiscence as the cure of souls in early old age. J Relig Health. (1981) 20:41–7. doi: 10.1007/BF01533286, PMID: 24311049

[11] Westerhof GJ Bohlmeijer ET. Celebrating fifty years of research and applications in reminiscence and life review: state of the art and new directions. J Aging Stud. (2014) 29:107–14. doi: 10.1016/j.jaging.2014.02.003, PMID: 24655678

[12] Ingersoll-Dayton B Kropf N Campbell R Parker M. A systematic review of dyadic approaches to reminiscence and life review among older adults. Aging Ment Health. (2019, 2019) 23:1074–85. 30596457 10.1080/13607863.2018.1555696

[13] Good Alice Wilson Clare Ancient Claire Sambhanthan Arunasalam. (2013). A proposal to support wellbeing in people with borderline personality disorder: applying reminiscent theory in a mobile app. Paper presented at The ACM conference on Designing Interactive Systems, Newcastle. (2013). doi: 10.48550/arXiv.1302.5200

[14] Lazar A Thompson H Demiris G. A systematic review of the use of technology for remi-niscence therapy. Health Educ Behav. (2014) 41:51S–61S. doi: 10.1177/1090198114537067 25274711 PMC4844844

[15] Jun ES Do JD. A study on the effectiveness of reminiscence therapy for the demented elderly: centered on the improvement of the cognition, memory, behavior problems and the activity of daily living. Health Welfare. (2004) 7:23–36. doi: 10.23948/kshw.2004.12.7.23

[16] Sprecher S Hampton AJ Heinzel HJ Felmlee D. Can I connect with both you and my social network? Access to network-salient communication technology and get-acquainted interactions. Comput Hum Behav. (2016) 62:423–32. doi: 10.1016/j.chb.2016.03.090

[17] Deterding S. Dixon D. Khaled R. Nacke L., “From game design elements to gamefulness: defining gamification”, Proceedings of the 15th international academic MindTrek conference: Envisioning future media environments, (2011), pp. 9–15.

[18] Huotari K. Hamari J., “Defining gamification: a service marketing perspective”, Proceedings of the 16th international academic MindTrek conference, (2012), pp. 17–22.

[19] Hamari J Koivisto J Sarsa H. Does gamification work? --a literature review of empirical studies on gamification//2014 47th Hawaii international conference on system sciences. (2014): 3025–3034.

[20] Cheng Y-F Cho S-Y Tang H-H. Memoir monopoly: improving rehabilitation activities for elderly people with dementia. User Exp Magaz. (2017) 17

[21] Cadamuro Alessia Visch Valentijn. (2013). ‘What remains?’: A persuasive story telling game. In Games for health. Springer, Wiesbaden, GER, 153–160

[22] Schöbel S Saqr M Janson A. Two decades of game concepts in digital learning environments – A bibliometric study and research agenda. Comp Educ. (2021) 173–196. doi: 10.1016/j.compedu.2021.104296

[23] Caponetto I Earp J Ott M. Gamification and education: a literature review. European conference on games based learning. Academic conferences international limited, (2014), 1: 50.

[24] De Schutter B. Never too old to play: the appeal of digital games to an older audience. Games Cult. (2011) 2:155–70.

[25] Allaire JC McLaughlin AC Trujillo A Whitlock LA LaPorte L Gandy M. Successful aging through digital games: socioemotional differences between older adult gamers and non-gamers. Comput Hum Behav. (2013) 29:1302–6.

[26] Osmanovic S Pecchioni L. Beyond entertainment: motivations and outcomes of video game playing by older adults and their younger family members. Games and. Culture. (2016) 11:130–49. doi: 10.1177/1555412015602819, PMID: 40297624

[27] Zhang Fan. (2018). Intergenerational play between young people and old family members: patterns, benefits, and challenges. In International conference on human aspects of IT for the aged population, 581–593. doi: 10.1007/978-3-319-92034-4_44

[28] De la Hera T Loos E Simons M Blom J. Benefits and factors influencing the design of intergenerational digital games: a systematic literature review. Societies. (2017) 7:18. doi: 10.3390/soc7030018

[29] Osmanovic Sanela Pecchioni Loretta L. (2019). Playing with words: the experience of self-disclosure in in-tergenerational gaming. In International conference on human-computer interaction, 189–203.

[30] CHO S-Y. Memoir monopoly: a rehabilitation game for elderly living with dementia. Geron. (2014) 13:2. doi: 10.4017/gt.2014.13.02.209.00

[31] Zhong S Lee C Foster MJ Bian J. Intergenerational communities: a systematic literature review of intergenerational interactions and older adults’ health-related outcomes. Soc Sci Med. (2020) 264:113374. doi: 10.1016/j.socscimed.2020.113374, PMID: 33017736

[32] Bertran Ferran Altarriba Segura Elena Márquez Duval Jared Isbister Katherine: Chasing play potentials: towards an increasingly situated and emergent approach to everyday play design[C]. Designing interactive systems conference, (2019): 1265–1277.

[33] Braun V Clarke V Hayfield N. ‘A starting point for your journey, not a map’: Nikki Hayfield in conversation with Virginia Braun and Victoria Clarke about thematic analysis. Qual Res Psychol. (2022) 19:424–45. doi: 10.1080/14780887.2019.1670765

[34] Lee S Oh H Shi C. Life review using a life metaphoric game to promote intergenerational communication. Proc. ACM Human-Comp Inter. (2020) 4:1–21. doi: 10.1145/3415169, PMID: 39076787

[35] Orwin L Kist A A Maxwell A D. Using gamification to create opportunities for engagement, collaboration and communication in a peer-to-peer environment for making and using remote access labs//2015 3rd experiment international conference (exp. at'15), (2015): 230–236.

[36] Killen A Macaskill A. Using a gratitude intervention to enhance well-being in older adults. J Happiness Stud. (2015) 16:947–64. doi: 10.1007/s10902-014-9542-3

[37] Zhang F Kaufman D Schell R Salgado G Seah ETW Jeremic J. Situated learning through intergenerational play between older adults and undergraduates. Int J Educ Technol High Educ. (2017) 14:16. doi: 10.1186/s41239-017-0055-0

[38] Rice Mark Cheong Yian Ling Ng Jamie Chua Puay Hoe Theng Yin-Leng. (2012). Co-creating games through intergenerational design workshops. In Proceedings of the designing interactive systems conference. 368–377.

[39] De Schutter B Julie AB. Digital games as a source of enjoyment in later life. Games Culture. (2016) 11:28–52. doi: 10.1177/1555412015594273

[40] De Schutter Bob Vanden Abeele Vero. (2010). Designing meaningful play within the psycho-social context of older adults. In Proceedings of the 3rd international conference on fun and games. 84–93.

[41] Vasconcelos LA Crilly N. Inspiration and fixation: questions, methods, findings, and challenges. Des Stud. (2016) 42:1–32. doi: 10.1016/j.destud.2015.11.001

[42] Vargas-Hernández A Robledo S Quiceno GR. Virtual teaching for online learning from the perspective of higher education: a bibliometric analysis[J]. J Scientometr Res. (2024) 13:406–18.

